# Brittle–Ductile Threshold in Lithium Disilicate under Sharp Sliding Contact

**DOI:** 10.1177/00220345241256279

**Published:** 2024-06-14

**Authors:** M. Bawazir, C.H. Lim, P. Arnés-Urgellés, M. Lu, H. Huang, Y. Zhang

**Affiliations:** 1 Department of Preventive and Restorative Sciences, School of Dental Medicine, University of Pennsylvania, Philadelphia, PA, USA; 2 Restorative Dentistry Department, Faculty of Dentistry, King Abdulaziz University, Jeddah, Saudi Arabia; 3 School of Mechanical and Mining Engineering, The University of Queensland, St Lucia, QLD, Australia; 4 School of Advanced Manufacturing, Sun Yat-sen University (Shenzhen Campus), China

**Keywords:** ductile grinding, lithia glass-ceramics, brittle−ductile transition, scratch load, strength degradation, subsurface damage

## Abstract

Computer-aided design (CAD)/computer-aided manufacturing (CAM) milling and handpiece grinding are critical procedures in the fabrication and adjustment of ceramic dental restorations. However, due to the formation of microfractures, these procedures are detrimental to the strength of ceramics. This study analyzes the damage associated with current brittle-regime grinding and presents a potential remedy in the application of a safer yet still efficient grinding regime known as “ductile-regime grinding.” Disc-shaped specimens of a lithium disilicate glass-ceramic material (IPS e.max CAD) were obtained by cutting and crystallizing the lithium metasilicate CAD/CAM blanks (the so-called blue blocks) following the manufacturer’s instructions. The discs were then polished to a 1 µm diamond suspension finish. Single-particle micro-scratch tests (*n* = 10) with a conical diamond indenter were conducted to reproduce basic modes of deformation and fracture. Key parameters such as coefficient of friction and penetration depth were recorded as a function of scratch load. Further, biaxial flexure strength tests (*n* = 6) were performed after applying various scratch loads to analyze their effects on ceramic strength. Scanning electron microscopy (SEM) and focused ion beam (FIB) were used to characterize surface and subsurface damage. Statistical analysis was performed using one-way analysis of variance and Tukey tests. While the SEM surface analysis of scratch tracks revealed the occurrence of both ductile and brittle removal modes, it failed to accurately determine the threshold load for the brittle–ductile transition. The threshold load for brittle–ductile transition was determined to be 70 mN based on FIB subsurface damage analyses in conjunction with strength degradation studies. Below 70 mN, the specimens exhibited neither strength degradation nor the formation of subsurface cracks. Determination of the brittle–ductile thresholds is significant because it sets a foundation for future research on the feasibility of implementing ductile-regime milling/grinding protocols for fabricating damage-free ceramic dental restorations.

## Introduction

In the age of digital dentistry, monolithic ceramic restorations are becoming increasingly popular (Sieper et al. 2017; Zhang and Lawn 2018; Schweiger et al. 2023; Cesar et al. 2024; Sulaiman et al. 2024). Lithium disilicate glass-ceramics (LDS) are at the forefront of this movement owing to their requisite mechanical and esthetic properties but also reasonable fabrication costs (Zhang et al. 2023). However, due to the brittle nature of glass-ceramics, the strength or resistance to failure of a restoration can quickly diminish if deep penetrating grinding cracks are present (Chen et al. 2020; Romanyk et al. 2020; May et al. 2022). Indeed, studies have shown that hand grinding dental ceramics using coarse (125 µm), fine (46 or 30 µm), and extra-fine (15 µm) diamond burs decreased the flexural strength of an LDS material (IPS e.max CAD, Ivoclar Vivadent) by 24% to 26% (Cadore-Rodrigues et al. 2021), and for various zirconia compositions, the reduction ranges from 29% to 32% for 3Y- and 4Y-PSZ and from 55% to 59% for 5Y-PSZ (Abdulmajeed et al. 2022). Remarkably, postgrind handpiece polishing using recommended dental polishing kits did not significantly improve their flexural strength (Cadore-Rodrigues et al. 2021; Abdulmajeed et al. 2022). This is not surprising since the literature has shown that grinding using 75 and 54 µm diamond discs produced cracks averaging 100 and 84 µm deep in IPS e.max CAD, 106 and 51 µm deep in Mark II porcelain, and 13 and 6 µm deep in Lava 3Y-TZP, respectively (Canneto et al. 2016; Curran et al. 2017). Thus, postgrind handpiece polishing of a ceramic restoration does not guarantee complete removal of deep damage.

It is well-established that current diamond bur grinding protocols can effectively remove material but can also introduce strength-limiting subsurface microcracks (Vila-Nova et al. 2020; Cadore-Rodrigues et al. 2021; Abdulmajeed et al. 2022). Subsequent polishing protocols introduce less damage but are time-consuming and ineffective in removing deep grinding cracks (Alao et al. 2017; Cadore-Rodrigues et al. 2021; Abdulmajeed et al. 2022). The latest advances in the mechanics of grinding demonstrated that by controlling process parameters such as applied load, grinding speed, abrasive particle size, and penetration depth, material can be effectively removed without incurring strength-degrading damage, a regime known as “ductile grinding” (Bifano et al. 1991; Huang et al. 2021). In this region of grinding, microcracks may still form within a ductile chip but not penetrate below the cut surface. Although progress has been made in noninvasive ductile grinding of brittle engineering materials such as single crystal semiconductors and amorphous glasses (Patten et al. 2004; Kovalchenko 2013; Antwi et al. 2018), ductile grinding of dental ceramics has never been achieved (Choudhary and Paul 2021; Patidar et al. 2021; Liu et al. 2022; Song et al. 2022). The fundamental micromechanisms of ductile removal in dental ceramics remain poorly understood (Huang et al. 2021; Lawn et al. 2021).

This study aims to understand the fundamental removal mechanisms in a representative dental LDS material. Since dental bur grinding involves multiple microcontact processes with individual diamond particles in the translation mode, the fundamental mechanisms of dental ceramic grinding can be most conveniently elucidated by single-particle diamond scratch tests. Our hypothesis is that brittle and ductile removal regimes can be produced on LDS by well-controlled ramping load scratch tests. The objective of this study is to precisely determine the threshold load for the brittle–ductile transition in an LDS material via direct observation of subsurface damage in conjunction with strength degradation studies. Once we gain a fundamental understanding of scratch damages and removal mechanisms in simple testing configurations, we will then introduce more complex testing configurations, such as multiparticle scratches, definitive grinding tests, and eventually, diamond bur grinding studies of anatomically correct dental prostheses.

## Theory

### Coefficient of Friction Function

Based on our experimental observations, the dependence of coefficient of friction (COF) on the normal ramping load in “sharp” diamond scratch of LDS may be best described by a power-law relation. Thus, we can conveniently write the COF expression, *µ*, as a function of normal load *P*_N_:

(1) $$\mu \;\;=\;\mu_{I}\;+\;\alpha \left(\mu_{F}\;-\;\mu_{I}\right){\left(P_{N\;}\;-\;P_{NI}\right)}^{m}\;\left(P_{I\;}\leq \;P\leq P_{F\;}\right)$$

where *µ*_I_ and *µ*_F_ and *P*_NI_ and *P*_NF_ represent the initial and final COF and normal scratch load, respectively. *m* is a dimensionless constant. *α* is a scale factor, which can be derived by rearranging equation 1:

(2) $$\alpha \;=\;\left(\mu \;-\;\mu_{I}\right)/\left[\left(\mu_{F}\;-\;\mu_{I}\right){\left(P_{N\;}\;-\;P_{NI}\right)}^{m}\right]$$

At *P*_N_ = *P*_NF_ and *µ* = *µ*_F_, we obtain

(3) $$\alpha \;=\;{1/\left(P_{NF\;}\;-\;\;P_{NI}\right)}^{m}$$

Thus, equation 1 can be rewritten in a generic form:

(4) $$\mu \;=\;\mu_{I}\;+\;\left(\mu_{F}\;-\;\mu_{I}\right){\left[\left(P_{N\;}\;-\;P_{NI}\right)/\left(P_{NF\;}\;-\;P_{NI}\right)\right]}^{m}$$

### Scratch Strength Degradation Analysis

The mechanics for strength degradation due to axial indentation have been well-established (Chantikul et al. 1981). Beyond the radial/median crack threshold, the dependence of ceramic strength *S* on indentation load *P* takes the form (Lawn and Cook 2012):

(5) $$S=T^{4/3}/\eta^{4/3}P^{1/3}$$

where *T* is the toughness of the ceramic. *η* = 0.88 is a dimensionless coefficient, calibrated over a wide range of ceramics (Chantikul et al. 1981).

Now we extend our analysis to the scratch test. The COF associated with a sliding indenter intensifies tensile stresses. Assume radial/median cracks form at the same tensile stress under axial loading while sliding, the critical normal load *P*_N_ for the onset of fracture in sliding can be related to the critical load *P* in axial loading (Lawn and Frank 1967; Ren and Zhang 2014).

(6) $$P={\left(1+k\mu \mu \right)}^{3}P_{N}$$

and

(7) k=3π(4+ν)/[8(1−2ν)]

where ν is the Poisson’s ratio of the ceramic.

## Experimental Methods

### Specimen Preparation

We selected one of the most widely used and well-characterized glass-ceramic materials in IPS e.max CAD (Ivoclar Vivadent). The partially crystallized lithium metasilicate blue blocks were shaped into cylinders of ~12 mm diameter using a diamond grit hole saw. The cylinders were then sliced into 1.8 mm thickness discs using a low-speed precision cutter with water irrigation (IsoMet, Buehler). The disc specimens were then crystallized at 840 °C in a dental vacuum furnace (Programat EP 5000, Ivoclar Vivadent) following the manufacturer’s specifications. Specimens were lap polished with successively finer grits (15, 9, 6, and 3 µm) down to a 1 µm diamond suspension finish, under continuous water irrigation (Ecomet 300, Buehler). The final dimension of the specimens was Ø12 ×1 mm.

### X-Ray Diffraction Analysis

The crystalline phases of the disc-shaped specimens were characterized by X-ray diffraction (XRD) analysis using a CuKα (λ = 1.5418 Å) source (MiniFlex 6G, Rigaku) operating at 40 kV/15 mA. Continuous scanning conditions were set at a fixed interval of 0.02° and a scan rate of 1° min^−1^ from 10° to 70°.

### Microscratch Test

On the polished specimens, a series of ramping load single-particle scratch tests (*n* = 10) were performed using a conical diamond indenter (*r* = 5 µm tip radius and 120° cone angle), at a sliding speed of *v* = 50 µm/s (PB1000, Nanovea). Loads were monotonically increased from 0 to 180 mN at a rate of *P.* = 10 mN/s. Thus, the scratch distance *L* is determined by the applied normal scratch load *P*_N_: *L* = (*P*_N_ – *P*_NI_)(*v*/*P.*). Data of scratch load, COF, and penetration depth as a function of sliding distance were recorded. Note that all current scratch tests were conducted in air to simulate chairside bur adjustments so as to avoid measurement errors in penetration depth and surface profile due to the interference of water with contact conditions between the indenter and ceramic surface. The ramping load scratch tests were performed to introduce surface and subsurface damage, for determining the threshold load and penetration depth for brittle–ductile transition.

### Biaxial Flexure Test

An additional 70 disc-shaped specimens (Ø12 × 1 mm) were prepared, and their top surface was polished down to a 1 µm diamond suspension finish. Sixteen specimens were set aside for intrinsic strength measurement (no scratch). The remaining 54 specimens underwent a constant load scratch with a “sharp” diamond indenter (*r* = 5 µm, *v* = 50 µm/s, *L* = 7000 µm) across the center of the polished surface, at loads of 40, 50, 60, 70, 80, 90, 130, 200, and 300 mN (*n* = 6). The specimens were then subjected to piston-on-3-ball biaxial flexure, with the scratched face loaded in tension. The crosshead speed was 1 mm/min. Biaxial strength *S* was calculated according to ISO 6872/2015. Finally, the fractured pieces of each specimen were reassembled and the previously scratched surfaces observed in an optical microscope to determine whether fracture was initiated at or away from the scratched site.

### Scanning Electron Microscopy

The morphology of the scratch grooves and surface crack/damage were examined using an environmental field emission scanning electron microscope (Quanta 250 FEG, Thermo Fisher Scientific). The acceleration voltage of the imaging beam was 10 kV and the working distance was 10 mm. The onset and intensity of the surface crack/damage corresponding to the scratch load, penetration depth, and COF were carefully recorded.

### Focused Ion Beam–Scanning Electron Microscopy

Subsurface damage was evaluated using a dual-beam plasma focused ion beam scanning electron microscope (FIB-SEM; Tescan S8252X Plasma FIB, Tescan). Specimens subjected to a ramping load single-pass scratch were coated with a thin iridium conductive film. A silicon wafer was then affixed onto the scratched surface using graphite conductive adhesive 154 to decrease the artifacts from the material redeposition during ion milling. FIB milling and polishing were used to create site-specific layer-by-layer excavation in a form of a series of boxes—50 µm (width) × 22 µm (height) × 80 µm (depth)—in the specimen just below the scratch plane. The acceleration voltage of the imaging beam was 2 kV, with a current of 100 pA and a dwell time of 1 µs/pixel. The same procedure was repeated on 3 ramping load scratches (*n* = 3).

### Statistical Analysis

The GraphPad Prism 9 program was used for statistical analysis. After confirming the normal distribution of variables, one-way analysis of variance and post hoc Tukey’s tests with multiple comparisons at a confidence level of 95% were performed to compare the strength degradation as a function of applied load to the intrinsic strength of LDS. A 20% drop in strength was deemed as the transition point. Based on our power analysis, it was determined that 6 samples per group would be sufficient to provide 80% power with an alpha of *P* < 0.05 and a beta of 0.2 for an effect size of 2.

## Results

**Figure 1.**
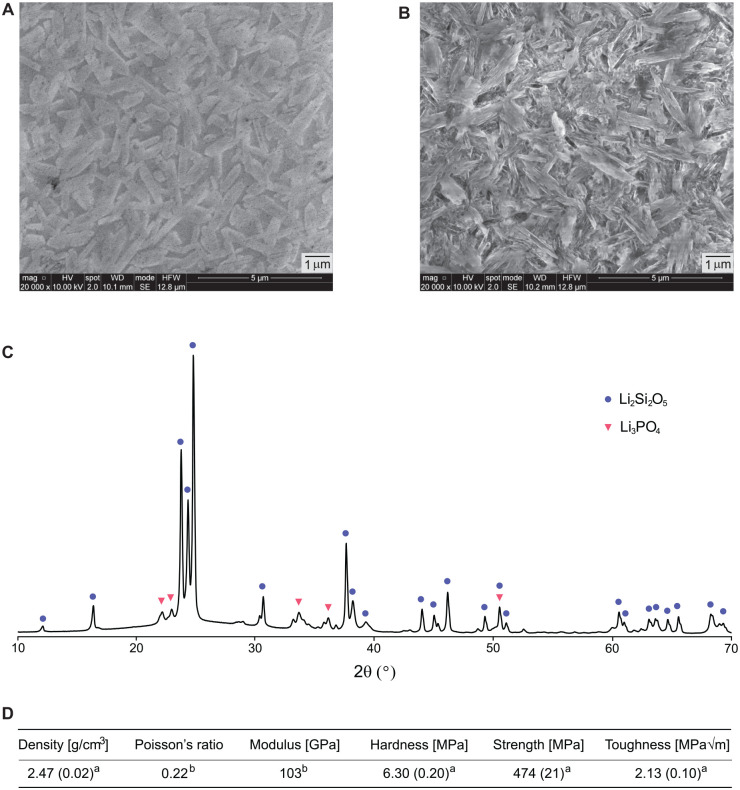
The microstructure, composition, and mechanical properties of lithium-disilicate glass-ceramic (LDS). Scanning electron microscopy images of (**A**) polished and (**B**) polished then etched (with 5% hydrofluoric acid for 20 s) sections, revealing elongated lithium disilicate crystallites. (**C**) X-ray diffraction analysis of the crystalline phases. (**D**) The physical and mechanical properties of LDS. ^a^Data measured in the authors’ laboratory on 1 µm diamond suspension polished surfaces: density determined by Archimedes’ method (*n* = 10), hardness from Vickers tests at 10 N (*n* = 5), strength from piston-on-3-ball biaxial flexure test with discs of 12 mm diameter and 1 mm thickness (*n* = 16), and toughness from the single-edge-V-notched-beam test (*n* = 8). ^b^Data from Laboure et al. (2022).

Figure 1 shows the microstructure, composition, and mechanical properties of LDS after crystallization firing at 840 °C. SEM images of polished (Fig. 1A) and polished and etched (etching was performed with 5% hydrofluoric acid for 20 s, Fig. 1B) surfaces revealed ~75 vol.% randomly oriented and elongated crystals (~1 to 2 µm long and ~0.4 µm wide in average) embedded in a glass matrix. The crystalline phases, based on XRD analysis, were composed primarily of a lithium disilicate (Li_2_Si_2_O_5_) phase, along with a minor lithium orthophosphate (Li_3_PO_4_) phase (Fig. 1C). The physical and mechanical properties of LDS are shown in Figure 1D.

**Figure 2.**
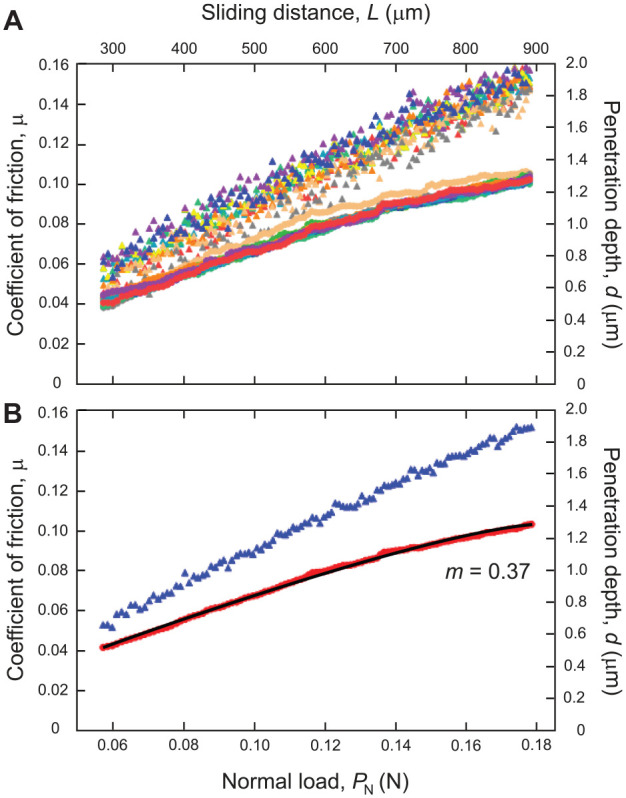
Plots of coefficient of friction (COF) and penetration depth as a function of normal scratch load and sliding distance derived from the experimental ramping load scratch data. (**A**) The circles are raw experimental data of COF, and the triangles are the penetration depth data of 10 scratches, where each shade represents an individual scratch data set. (**B**) The red circles are the average data of COF, and the blue triangles are the average penetration depth data for 10 scratches. The solid black curve is a closed-form equation (equation 4) fit to the average COF data.

Figure 2 shows data of COF (circles) and penetration depth (triangles) as a function of normal scratch load and sliding distance (*n* = 10). Figure 2A shows the raw experimental data of 10 scratches (each shade represents an individual scratch), whereas Figure 2B depicts the average data of all 10 scratches. The dependence of penetration depth *d* on *P*_N_ and *L* follows a linear relationship (Fig. 2B). However, the COF *µ* correlates to *P*_N_ in a power-law function (equation 4) with *m* = 0.37 (Fig. 2B). Note the *m* value was obtained by fitting the average COF data with equation 4 (see solid black curve in Fig. 2B), using SigmaPlot 12 software.

**Figure 3.**
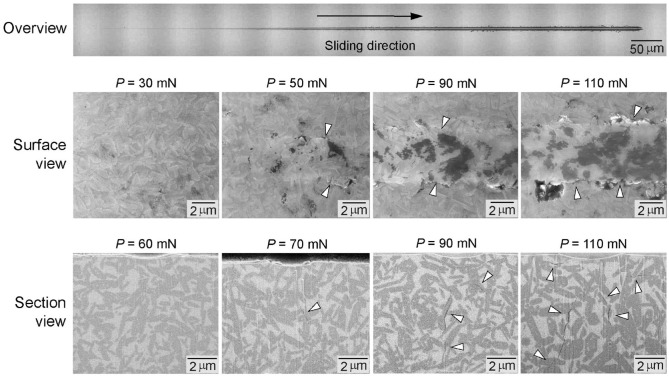
Microscopy analyses of damage sustained in the lithium-disilicate glass-ceramic (LDS) following a ramping load scratch test. (Top) Optical overview of a scratch. (Middle) Scanning electron microscopy (SEM) surface view of areas on a scratch track corresponding to *P*_N_ = 30, 50, 90, and 110 mN, showing regions with distinct removal modes: ductile, brittle, and the transition from ductile to brittle. Arrows indicate lateral cracks at the edges of the scratch track. (Bottom) Focused ion beam–SEM analysis of subsurface damage under the scratch path revealed that median cracks formed at approximately 70 mN, as indicated by the white arrow. The depth and intensity of subsurface cracks increased with the scratch load, as indicated by white arrows.

Figure 3 shows microscopy analyses of damage sustained in the LDS following a ramping load scratch test. Optical montage images show an overview of a scratch groove in which the load monotonically increases along the sliding direction (top panel). The SEM examination revealed three regions with distinct removal modes: ductile, brittle, and the transition from ductile to brittle. The absence of surface crack formation and smooth edges of the scratch groove under a low load <30 mN indicates pure plastic flow at the ductile region. The first incidence of lateral crack at the scratch boundaries at a load of about 50 mN suggests that a combination of plastic flow and microfracture would be the removal mode. However, after that, the frequency and intensity of the surface cracks increase with increasing scratching load, making it challenging to precisely determine the threshold load at which the removal mode transitioned from ductile to brittle (middle row). It is important to emphasize that the threshold load for the brittle–ductile transition, in which the flexural strength of the LDS begins to reduce due to the formation of substantial subsurface cracks, is unknown from surface observation. FIB-SEM subsurface damage analyses revealed that median cracks formed at approximately 70 mN (bottom row). The depth of these median cracks increased with the normal scratch load *P*_N_.

**Figure 4.**
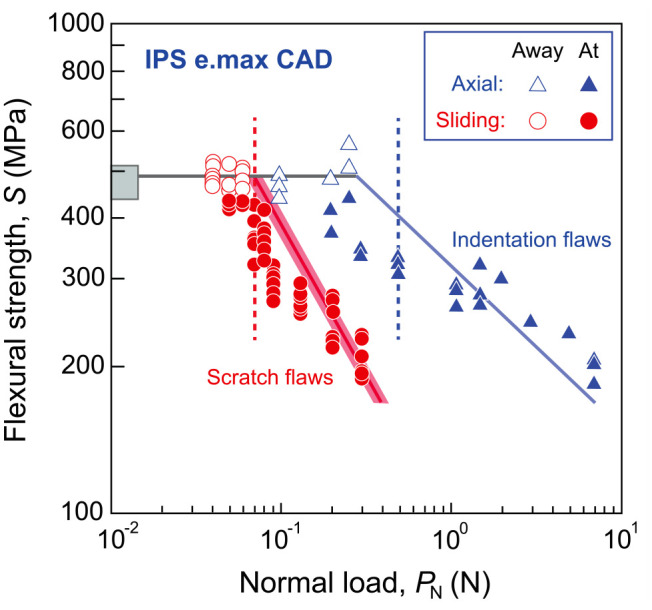
Plots of biaxial flexural strength as a function of scratch and indentation loads. The box on the left vertical axis shows the mean and standard deviations for the intrinsic biaxial flexural strength *S* obtained from polished samples without scratch or indentation (*n* = 16). Red circles represent flexural strength *S* versus normal scratch load *P*_N_ (*n* = 6). Filled symbols represent failure origins at scratch paths, and unfilled symbols show failures away from these paths. Blue triangles represent the flexural strength *S* versus indentation load plot from a previous study (Alves et al. 2022). Again, filled symbols represent failure origins at indentation sites, whereas unfilled symbols show failures away from these sites. The solid blue slope to the right is the theoretical prediction of the dependence of *S* on indentation load *P* (Alves et al. 2022), whereas the bold red slope represents the prediction of *S* versus normal scratch load *P*_N_ with 90% confidence bounds using equations 5 to 7. Vertical dashed lines are the predicted 20% reduction in the strength of lithium disilicate glass-ceramics (LDS) subjected to controlled scratch (red) and indentation (blue) relative to its intrinsic strength *S*.

A detailed flexural strength *S* versus normal scratch load *P*_N_ plot (red circles) is shown in Figure 4. For comparison, the flexural strength *S* versus Vickers indentation load plot (blue triangles) from a previous study (Alves et al. 2022) is also included. The solid blue slope to the right is the theoretical prediction of the dependence of *S* on indentation load *P* using equation 5 with material property data in Figure 1D, whereas the bold red slope represents the prediction of *S* versus normal scratch load *P*_N_ with 90% confidence bounds using equations 5 to 7. As can be seen, the predictions lie within the scatter of experimental data. Vertical dashed lines designate the predicted 20% reduction in *S* of LDS relative to its intrinsic *S* (474 ± 21 MPa) obtained from polished samples without scratch or indentation. At the lower loads of 40, 50, and 60 mN, *S* of the scratched specimens did not differ significantly relative to the intrinsic *S* (*P* < 0.05). However, at *P*_N_ = 70 mN, a ~20% drop in *S* (368.5 ± 35.50 MPa) was detected, indicating that 70 mN is the critical load below which the material removal falls under the ductile grinding regime. Above 70 mN, *S* continued to decrease as *P*_N_ increased.

**Figure 5.**
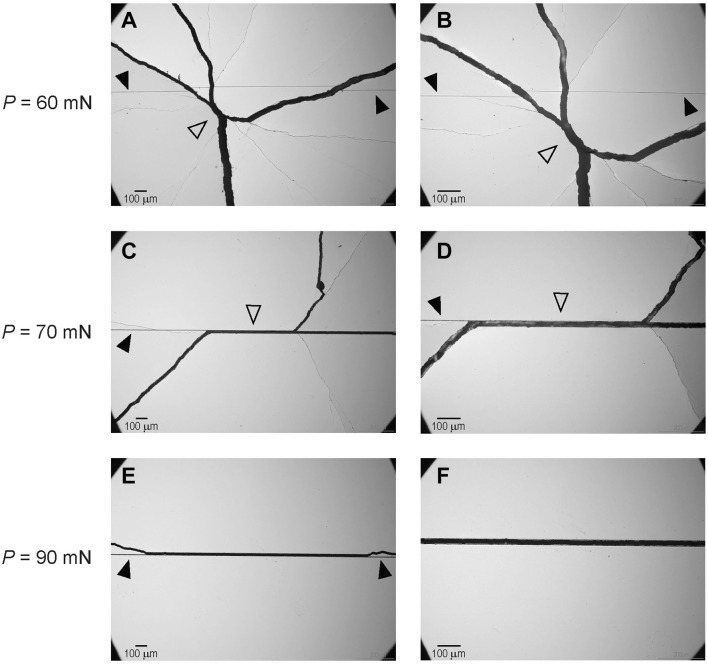
Fractographic analyses of fractured samples following the biaxial flexural strength *S* test under an optical microscope. (**A**, **B**) Images of fractured samples scratched with *P*_N_ ≤ 60 mN, where fracture paths did not pass the scratch groove, suggesting that failure originated from intrinsic microstructural flaws. (**C–F**) Images of fractured samples scratched with *P*_N_ ≥ 70 mN. The fracture paths passed through the scratch grooves, confirming that failure primarily originated from subsurface cracks below the scratch groove. Open arrows indicate the fracture path, while solid arrows indicate the scratch groove.

Following the biaxial flexural *S* test, fractured specimens were reassembled for fractography analyses under the optical microscope (Fig. 5). For *P*_N_ ≤ 60 mN, fracture paths (indicated by open arrows) did not pass the scratch groove (indicated by solid arrows), suggesting that failure originated from intrinsic microstructural flaws. However, for *P*_N_ ≥ 70 mN, the fracture paths passed through the scratch grooves, confirming that failure primarily originated from subsurface cracks below the scratch groove.

## Discussion

The objective of this study was to determine the brittle–ductile threshold in sharp diamond tip scratching of a widely used dental LDS material. Distinct surface damage modes and material removal mechanisms were observed in response to a ramping load scratch test. During this process, the relationship between the penetration depth and the ramping load followed a linear dependence, while the dependence of the COF on the load could be more accurately characterized by a power-law relationship. Although the ductile and brittle regions can be clearly observed via the surface SEM examination, the actual brittle–ductile threshold cannot be determined based solely on surface observations. These findings suggest that LDS glass-ceramics can undergo a transition from ductile to brittle removal mechanism; however, they do not provide information on brittle–ductile thresholds. To date, the experimental determination of brittle–ductile transitions in dental glass-ceramics using sharp diamond scratches has been based on surface damage observations without any subsurface damage evaluation and/or strength degradation validation (Yang et al. 2019; Li 2020; Kuo Lu 2023). Thus, the reported brittle–ductile thresholds lack a physical base since the formation of surface cracks does not necessarily imply that the strength of the glass-ceramic material is jeopardized.

Cracks induced by elastic/plastic contacts can be categorized into two types: those that form on symmetry median planes containing the load axis (median/radial cracks) and those that form laterally on planes closely parallel to the specimen surface (lateral cracks) (Lawn et al. 1980; Marshall et al. 1982). Surface damage and lateral cracks caused by grinding are responsible for material removal, whereas subsurface median/radial cracks are liable for strength degradation in brittle ceramics (Lawn et al. 1980; Zhang et al. 2004). In the current strength degradation study, the application of a 70 mN scratch load results in a 20% reduction in strength of LDS relative to the control. FIB-SEM analysis revealed a median crack beneath the scratch plan at 70 mN. Note that there was no subsurface crack formation when a scratch load of 60 mN or below was applied (Fig. 4). When the applied load was increased to 90 mN, the intensity of the subsurface damages amplified, as we observed several lateral cracks and a prominent median crack penetrated deep into the material subsurface. These findings align with our strength degradation experiments (Fig. 4), suggesting that the threshold load required for material removal to fall under the ductile grinding regime while maintaining the strength of LDS is 70 mN.

We have applied the indentation theory of sliding contacts to predict the brittle–ductile threshold as well as the dependence of flexural strength on the normal scratch load in an LDS material. It is well-established that in axial loading with a “sharp” indenter, indentation cracks begin to dominate the intrinsic flaw population beyond the radial/median crack threshold (Alves et al. 2022). In this brittle region, ceramic strength continues to decline as the indentation load increases, following a negative linear relationship on a logarithmic plot (equation 5). At the same time, the ceramic material retains its intrinsic strength while the indentations remain in the subthreshold ductile zone. By incorporating the COF function associated with the sliding contact, we can then predict the critical normal load *P*_N_ for the onset of fracture in sliding contact based on the critical load *P* in axial loading. Finally, the strength degradation of LDS under sliding contact can be predicted (equations 5 to 7). As can be seen in Figure 4, our theoretical prediction agrees well with the experimental results.

We acknowledge that the scratch tests performed in this study were conducted under dry conditions to ensure precise measurements of penetration depth. Although the single-particle scratch provides critical information on threshold load and penetration depth concerning brittle–ductile transition, it is only a first step toward the implementation of ductile-regime grinding in a clinical setting. Controlled multiparticle grinding studies in conjunction with subsurface damage evaluations and strength degradation analyses are required for establishing ductile-regime grinding of lithia glass-ceramics. The extension of ductile-grinding studies to other classes of dental ceramics is needed. The ultimate goal is to fabricate ceramic restorations free of strength-limiting damage through the implementation of ductile-grinding technology.

## Conclusions

- Lithium disilicate glass-ceramics underwent a transition from ductile to brittle removal as the scratch load increases.
- The threshold of the brittle–ductile transition cannot be determined based solely on surface damage observations.
- The threshold of ductile grinding below which the strength of ceramics is preserved can be identified by subsurface damage evaluations in conjunction with strength degradation studies.
- The critical load for the onset of strength degradation under sliding contact can be related to well-established axial load indentation theory with a defined COF function.

## Author Contributions

M. Bawazir, contributed to design, data acquisition, analysis, and interpretation, drafted and critically revised the manuscript; C.H. Lim, contributed to data acquisition and analysis, critically revised the manuscript; P. Arnés-Urgellés, contributed to data analysis, critically revised the manuscript; M. Lu, contributed to data interpretation, critically revised the manuscript; H. Huang, contributed to design, data interpretation, critically revised the manuscript; Y. Zhang, contributed to conception, design, data acquisition, analysis, and interpretation, drafted and critically revised the manuscript. All authors gave their final approval and agree to be accountable for all aspects of work.
